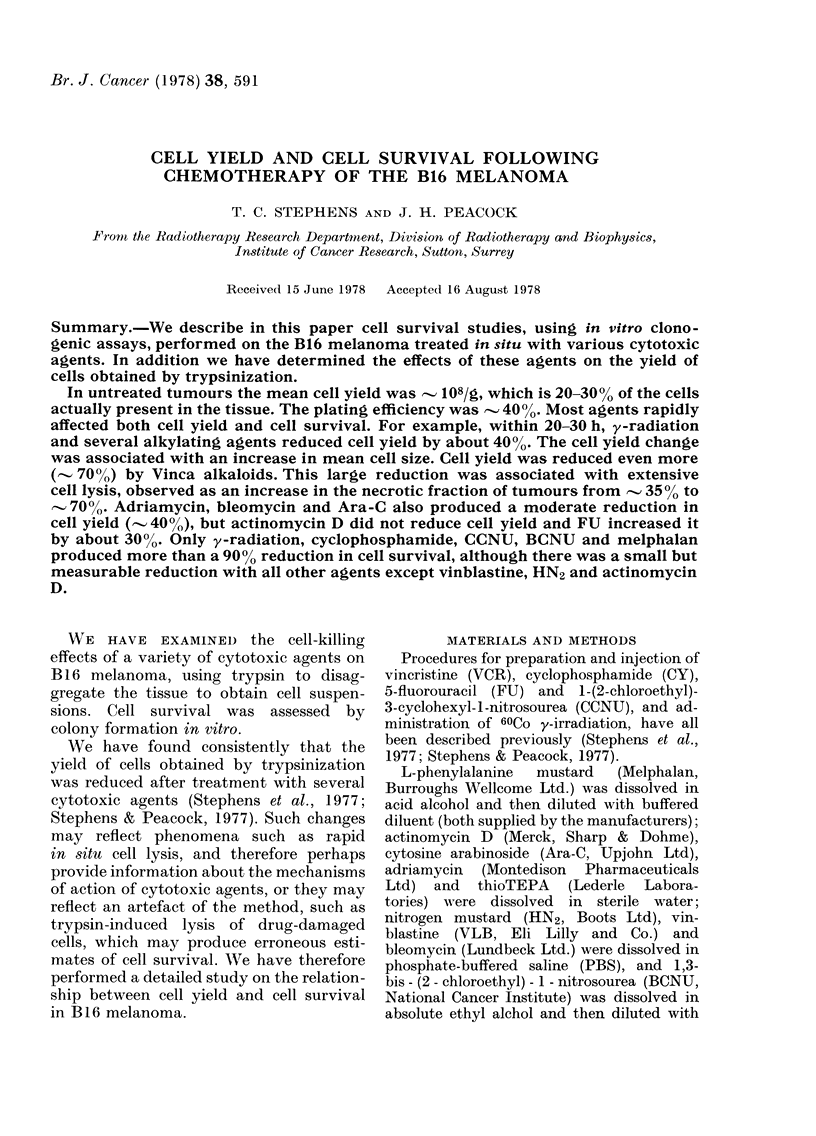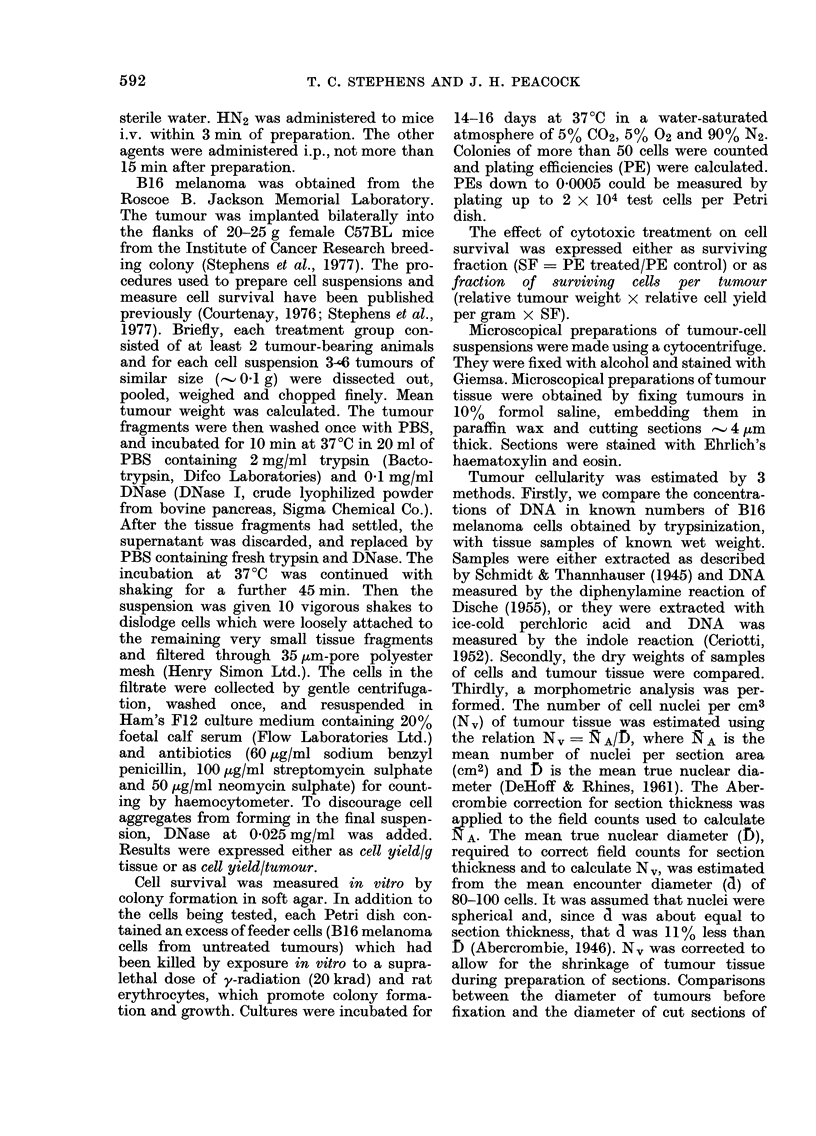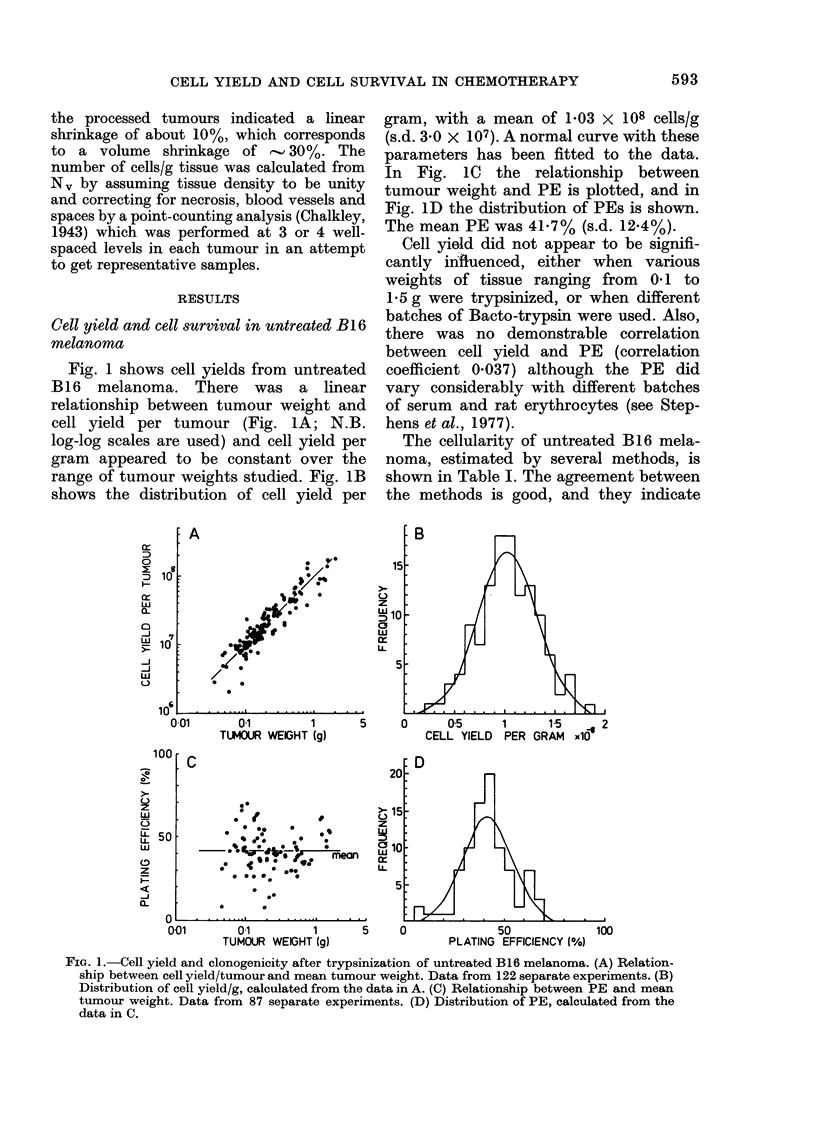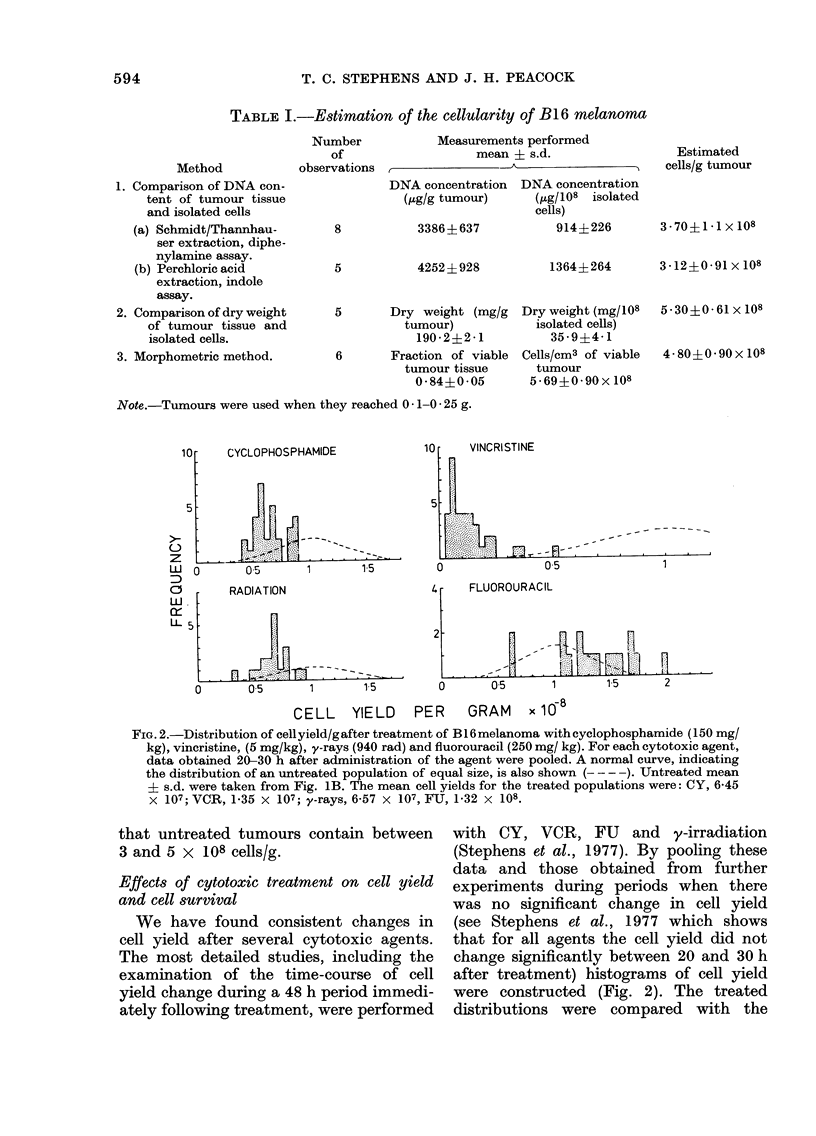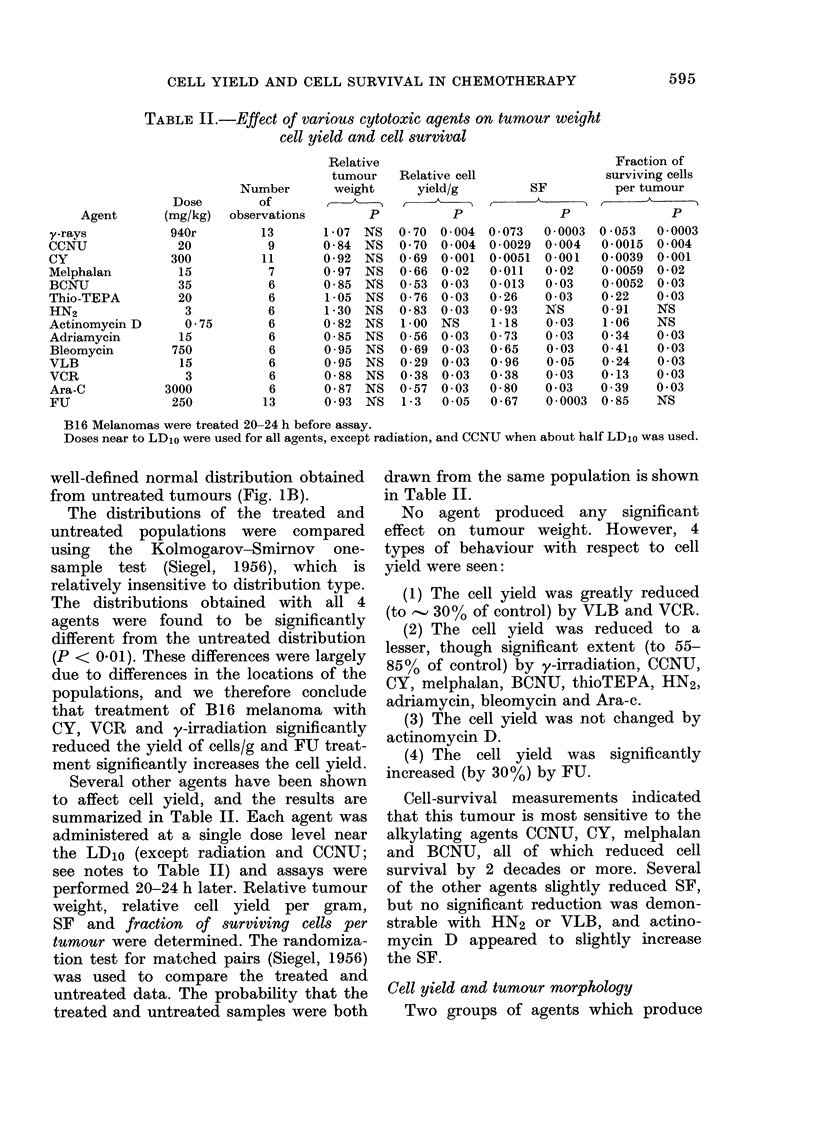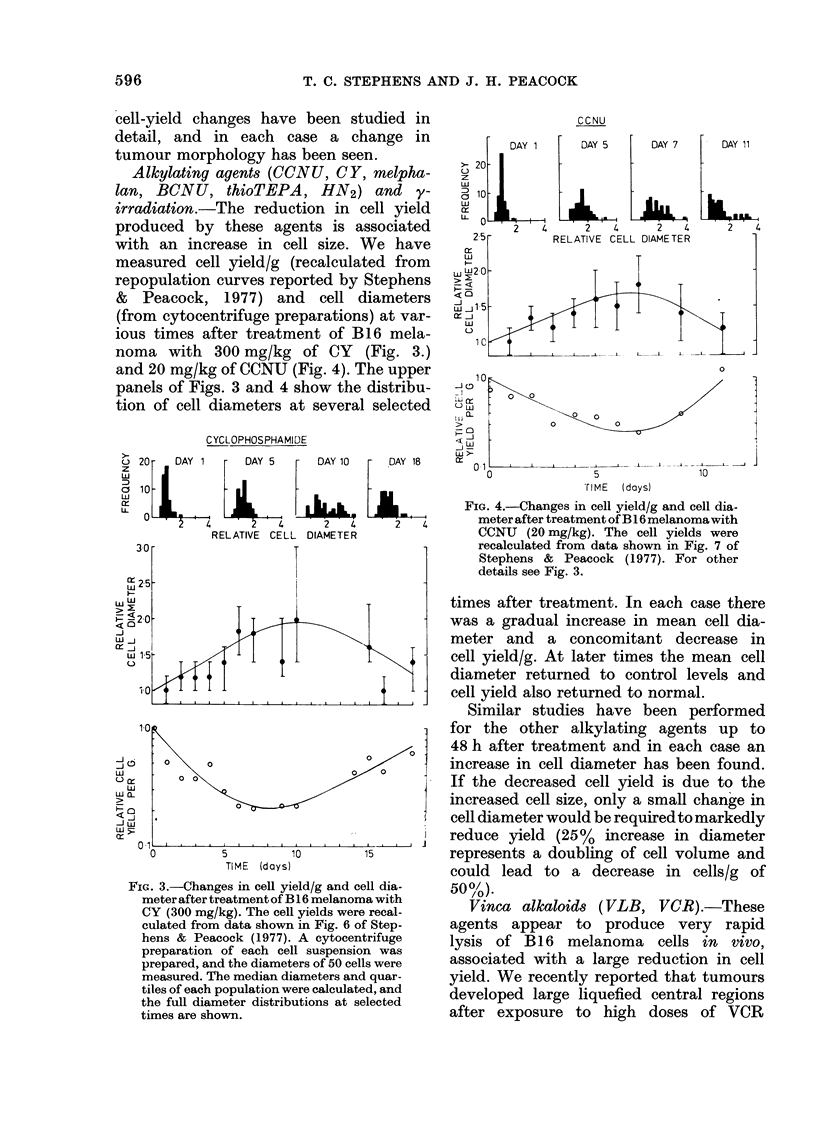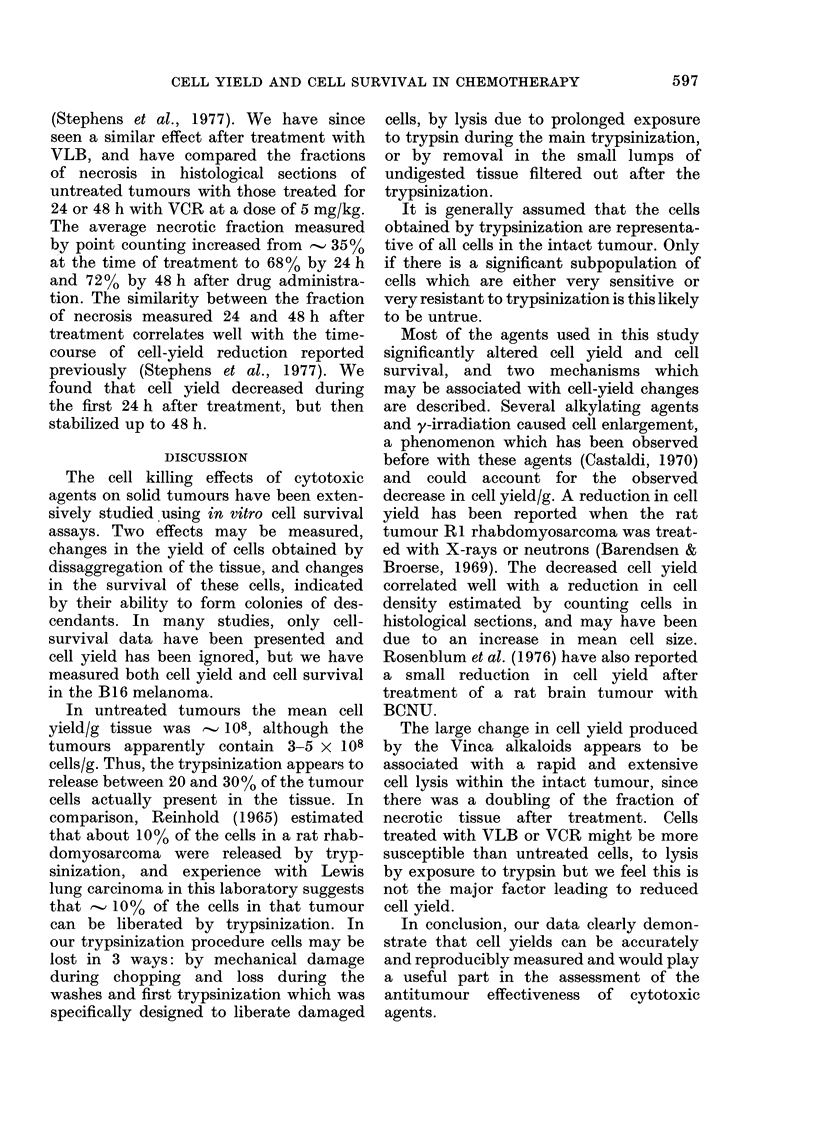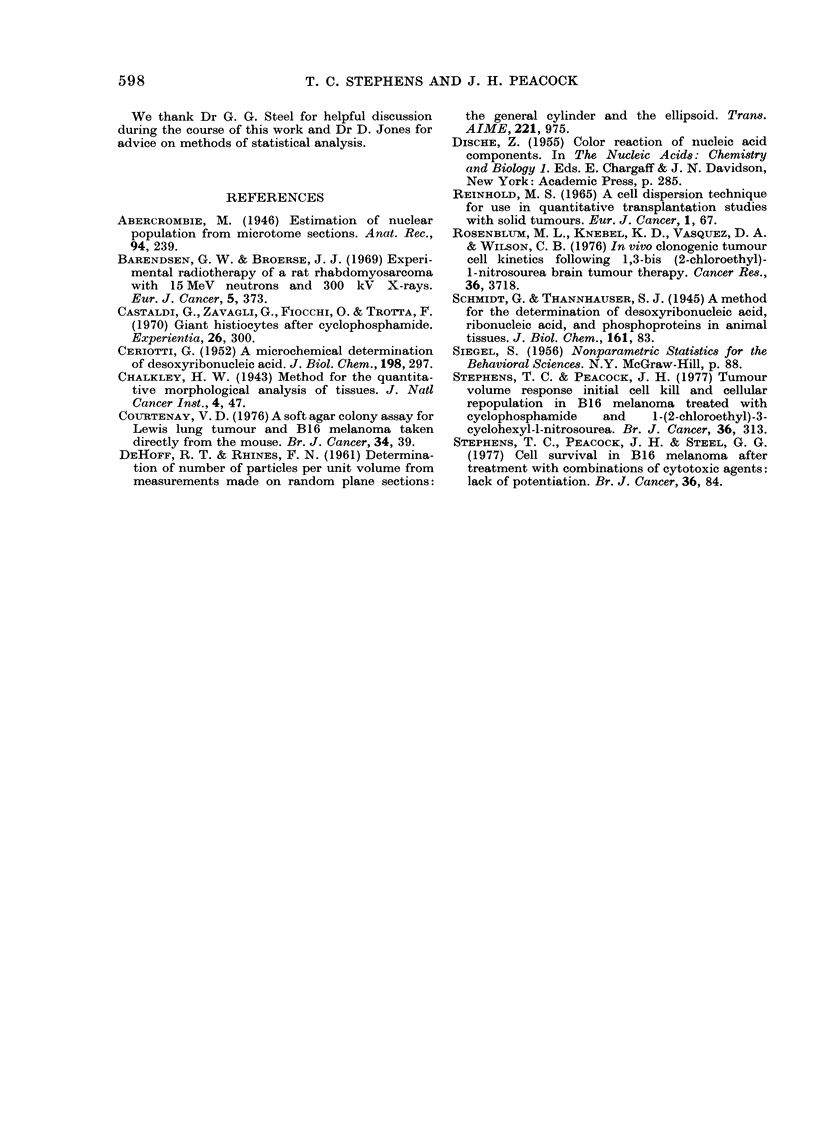# Cell yield and cell survival following chemotherapy of the B16 melanoma.

**DOI:** 10.1038/bjc.1978.254

**Published:** 1978-11

**Authors:** T. C. Stephens, J. H. Peacock

## Abstract

We describe in this paper cell survival studies, using in vitro clonogenic assays, performed on the B16 melanoma treated in situ with various cytotoxic agents. In addition we have determined the effects of these agents on the yield of cells obtained by trypsinization. In untreated tumours the mean cell yield was approximately 10(8)/g, which is 20--30% of the cells actually present in the tissue. The plating efficiency was approximately 40%. Most agents rapidly affected both cell yield and cell survival. For example, within 20--30 h, gamma-radiation and several alkylating agents reduced cell yield by about 40%. The cell yield change was associated with an increase in mean cell size. Cell yield was reduced even more (approximately 70%) by Vinca alkaloids. This large reduction was associated with extensive cell lysis, observed as an increase in the necrotic fraction of tumours from approximately 35% to approximately 70%. Adriamycin, bleomycin and Ara-C also produced a moderate reduction in cell yield (approximately 40%), but actinomycin D did not reduce cell yield and FU increased it by about 30%. Only gamma-radiation, cyclophosphamide, CCNU, BCNU and melphalan produced more than a 90% reduction in cell survival, although there was a small but measurable reduction with all other agents except vinblastine, HN2 and actinomycin D.


*Br. J. Cancer* (1978) **38**, 591

### CELL YIELD AND CELL SURVIVAL FOLLOWING CHEMOTHERAPY OF THE B16 MELANOMA

T. C. STEPHENS AND J. H. PEACOCK

*From the Radiotherapy Research Department, Division of Radiotherapy and Biophysics, Institute of Cancer Research, Sutton, Surrey*

Received 15 June 1978 Accepted 16 August 1978

**Summary.—We describe in this paper cell survival studies, using *in vitro* clonogenic assays, performed on the B16 melanoma treated *in situ* with various cytotoxic agents. In addition we have determined the effects of these agents on the yield of cells obtained by trypsinization.**

**In untreated tumours the mean cell yield was ~ $10^8$/g, which is 20–30% of the cells actually present in the tissue. The plating efficiency was ~ 40%. Most agents rapidly affected both cell yield and cell survival. For example, within 20–30 h, $\gamma$-radiation and several alkylating agents reduced cell yield by about 40%. The cell yield change was associated with an increase in mean cell size. Cell yield was reduced even more (~ 70%) by Vinca alkaloids. This large reduction was associated with extensive cell lysis, observed as an increase in the necrotic fraction of tumours from ~ 35% to ~ 70%. Adriamycin, bleomycin and Ara-C also produced a moderate reduction in cell yield (~ 40%), but actinomycin D did not reduce cell yield and FU increased it by about 30%. Only $\gamma$-radiation, cyclophosphamide, CCNU, BCNU and melphalan produced more than a 90% reduction in cell survival, although there was a small but measurable reduction with all other agents except vinblastine, $HN_2$ and actinomycin D.**

WE HAVE EXAMINED the cell-killing effects of a variety of cytotoxic agents on B16 melanoma, using trypsin to disaggregate the tissue to obtain cell suspensions. Cell survival was assessed by colony formation *in vitro*.

We have found consistently that the yield of cells obtained by trypsinization was reduced after treatment with several cytotoxic agents (Stephens *et al.*, 1977; Stephens & Peacock, 1977). Such changes may reflect phenomena such as rapid *in situ* cell lysis, and therefore perhaps provide information about the mechanisms of action of cytotoxic agents, or they may reflect an artefact of the method, such as trypsin-induced lysis of drug-damaged cells, which may produce erroneous estimates of cell survival. We have therefore performed a detailed study on the relationship between cell yield and cell survival in B16 melanoma.

### MATERIALS AND METHODS

Procedures for preparation and injection of vincristine (VCR), cyclophosphamide (CY), 5-fluorouracil (FU) and 1-(2-chloroethyl)-3-cyclohexyl-1-nitrosourea (CCNU), and administration of $^{60}$Co $\gamma$-irradiation, have all been described previously (Stephens *et al.*, 1977; Stephens & Peacock, 1977).

L-phenylalanine mustard (Melphalan, Burroughs Wellcome Ltd.) was dissolved in acid alcohol and then diluted with buffered diluent (both supplied by the manufacturers); actinomycin D (Merck, Sharp & Dohme), cytosine arabinoside (Ara-C, Upjohn Ltd), adriamycin (Montedison Pharmaceuticals Ltd) and thioTEPA (Lederle Laboratories) were dissolved in sterile water; nitrogen mustard ($HN_2$, Boots Ltd), vinblastine (VLB, Eli Lilly and Co.) and bleomycin (Lundbeck Ltd.) were dissolved in phosphate-buffered saline (PBS), and 1,3-bis - (2 - chloroethyl) - 1 - nitrosourea (BCNU, National Cancer Institute) was dissolved in absolute ethyl alchol and then diluted with sterile water. $HN_2$ was administered to mice i.v. within 3 min of preparation. The other agents were administered i.p., not more than 15 min after preparation.

B16 melanoma was obtained from the Roscoe B. Jackson Memorial Laboratory. The tumour was implanted bilaterally into the flanks of 20–25 g female C57BL mice from the Institute of Cancer Research breeding colony (Stephens *et al.*, 1977). The procedures used to prepare cell suspensions and measure cell survival have been published previously (Courtenay, 1976; Stephens *et al.*, 1977). Briefly, each treatment group consisted of at least 2 tumour-bearing animals and for each cell suspension 3–6 tumours of similar size ($\sim$0·1 g) were dissected out, pooled, weighed and chopped finely. Mean tumour weight was calculated. The tumour fragments were then washed once with PBS, and incubated for 10 min at 37°C in 20 ml of PBS containing 2 mg/ml trypsin (Bacto-trypsin, Difco Laboratories) and 0·1 mg/ml DNase (DNase I, crude lyophilized powder from bovine pancreas, Sigma Chemical Co.). After the tissue fragments had settled, the supernatant was discarded, and replaced by PBS containing fresh trypsin and DNase. The incubation at 37°C was continued with shaking for a further 45 min. Then the suspension was given 10 vigorous shakes to dislodge cells which were loosely attached to the remaining very small tissue fragments and filtered through 35 $\mu$m-pore polyester mesh (Henry Simon Ltd.). The cells in the filtrate were collected by gentle centrifugation, washed once, and resuspended in Ham's F12 culture medium containing 20% foetal calf serum (Flow Laboratories Ltd.) and antibiotics (60 $\mu$g/ml sodium benzyl penicillin, 100 $\mu$g/ml streptomycin sulphate and 50 $\mu$g/ml neomycin sulphate) for counting by haemocytometer. To discourage cell aggregates from forming in the final suspension, DNase at 0·025 mg/ml was added. Results were expressed either as *cell yield/g* tissue or as *cell yield/tumour*.

Cell survival was measured *in vitro* by colony formation in soft agar. In addition to the cells being tested, each Petri dish contained an excess of feeder cells (B16 melanoma cells from untreated tumours) which had been killed by exposure *in vitro* to a supralethal dose of $\gamma$-radiation (20 krad) and rat erythrocytes, which promote colony formation and growth. Cultures were incubated for 14–16 days at 37°C in a water-saturated atmosphere of 5% $CO_2$, 5% $O_2$ and 90% $N_2$. Colonies of more than 50 cells were counted and plating efficiencies (PE) were calculated. PEs down to 0·0005 could be measured by plating up to $2 \times 10^4$ test cells per Petri dish.

The effect of cytotoxic treatment on cell survival was expressed either as surviving fraction (SF = PE treated/PE control) or as *fraction of surviving cells per tumour* (relative tumour weight $\times$ relative cell yield per gram $\times$ SF).

Microscopical preparations of tumour-cell suspensions were made using a cytocentrifuge. They were fixed with alcohol and stained with Giemsa. Microscopical preparations of tumour tissue were obtained by fixing tumours in 10% formol saline, embedding them in paraffin wax and cutting sections $\sim$4 $\mu$m thick. Sections were stained with Ehrlich's haematoxylin and eosin.

Tumour cellularity was estimated by 3 methods. Firstly, we compare the concentrations of DNA in known numbers of B16 melanoma cells obtained by trypsinization, with tissue samples of known wet weight. Samples were either extracted as described by Schmidt & Thannhauser (1945) and DNA measured by the diphenylamine reaction of Dische (1955), or they were extracted with ice-cold perchloric acid and DNA was measured by the indole reaction (Ceriotti, 1952). Secondly, the dry weights of samples of cells and tumour tissue were compared. Thirdly, a morphometric analysis was performed. The number of cell nuclei per cm$^3$ ($N_V$) of tumour tissue was estimated using the relation $N_V = \bar{N}_A/\bar{D}$, where $\bar{N}_A$ is the mean number of nuclei per section area (cm$^2$) and $\bar{D}$ is the mean true nuclear diameter (DeHoff & Rhines, 1961). The Abercrombie correction for section thickness was applied to the field counts used to calculate $\bar{N}_A$. The mean true nuclear diameter ($\bar{D}$), required to correct field counts for section thickness and to calculate $N_V$, was estimated from the mean encounter diameter ($\bar{d}$) of 80–100 cells. It was assumed that nuclei were spherical and, since $\bar{d}$ was about equal to section thickness, that $\bar{d}$ was 11% less than $\bar{D}$ (Abercrombie, 1946). $N_V$ was corrected to allow for the shrinkage of tumour tissue during preparation of sections. Comparisons between the diameter of tumours before fixation and the diameter of cut sections of the processed tumours indicated a linear shrinkage of about 10%, which corresponds to a volume shrinkage of $\sim$30%. The number of cells/g tissue was calculated from $N_V$ by assuming tissue density to be unity and correcting for necrosis, blood vessels and spaces by a point-counting analysis (Chalkley, 1943) which was performed at 3 or 4 well-spaced levels in each tumour in an attempt to get representative samples.

### RESULTS

### *Cell yield and cell survival in untreated B16 melanoma*

Fig. 1 shows cell yields from untreated B16 melanoma. There was a linear relationship between tumour weight and cell yield per tumour (Fig. 1A; N.B. log-log scales are used) and cell yield per gram appeared to be constant over the range of tumour weights studied. Fig. 1B shows the distribution of cell yield per gram, with a mean of $1{\cdot}03 \times 10^8$ cells/g (s.d. $3{\cdot}0 \times 10^7$). A normal curve with these parameters has been fitted to the data. In Fig. 1C the relationship between tumour weight and PE is plotted, and in Fig. 1D the distribution of PEs is shown. The mean PE was 41·7% (s.d. 12·4%).

Cell yield did not appear to be significantly influenced, either when various weights of tissue ranging from 0·1 to 1·5 g were trypsinized, or when different batches of Bacto-trypsin were used. Also, there was no demonstrable correlation between cell yield and PE (correlation coefficient 0·037) although the PE did vary considerably with different batches of serum and rat erythrocytes (see Stephens *et al.*, 1977).

The cellularity of untreated B16 melanoma, estimated by several methods, is shown in Table I. The agreement between the methods is good, and they indicate

FIG. 1.—Cell yield and clonogenicity after trypsinization of untreated B16 melanoma. (A) Relationship between cell yield/tumour and mean tumour weight. Data from 122 separate experiments. (B) Distribution of cell yield/g, calculated from the data in A. (C) Relationship between PE and mean tumour weight. Data from 87 separate experiments. (D) Distribution of PE, calculated from the data in C.

TABLE I.—*Estimation of the cellularity of B16 melanoma*

| Method | Number of observations | Measurements performed mean ± s.d. | | Estimated cells/g tumour |
|---|---|---|---|---|
| 1. Comparison of DNA content of tumour tissue and isolated cells | | DNA concentration ($\mu$g/g tumour) | DNA concentration ($\mu$g/$10^8$ isolated cells) | |
| (a) Schmidt/Thannhauser extraction, diphenylamine assay. | 8 | 3386±637 | 914±226 | $3 \cdot 70 \pm 1 \cdot 1 \times 10^8$ |
| (b) Perchloric acid extraction, indole assay. | 5 | 4252±928 | 1364±264 | $3 \cdot 12 \pm 0 \cdot 91 \times 10^8$ |
| 2. Comparison of dry weight of tumour tissue and isolated cells. | 5 | Dry weight (mg/g tumour) 190·2±2·1 | Dry weight (mg/$10^8$ isolated cells) 35·9±4·1 | $5 \cdot 30 \pm 0 \cdot 61 \times 10^8$ |
| 3. Morphometric method. | 6 | Fraction of viable tumour tissue 0·84±0·05 | Cells/cm³ of viable tumour $5 \cdot 69 \pm 0 \cdot 90 \times 10^8$ | $4 \cdot 80 \pm 0 \cdot 90 \times 10^8$ |

*Note.*—Tumours were used when they reached 0·1–0·25 g.

FIG. 2.—Distribution of cell yield/g after treatment of B16 melanoma with cyclophosphamide (150 mg/kg), vincristine, (5 mg/kg), $\gamma$-rays (940 rad) and fluorouracil (250 mg/kg). For each cytotoxic agent, data obtained 20–30 h after administration of the agent were pooled. A normal curve, indicating the distribution of an untreated population of equal size, is also shown (– – – –). Untreated mean ± s.d. were taken from Fig. 1B. The mean cell yields for the treated populations were: CY, $6 \cdot 45 \times 10^7$; VCR, $1 \cdot 35 \times 10^7$; $\gamma$-rays, $6 \cdot 57 \times 10^7$, FU, $1 \cdot 32 \times 10^8$.

that untreated tumours contain between 3 and 5 × $10^8$ cells/g.

*Effects of cytotoxic treatment on cell yield and cell survival*

We have found consistent changes in cell yield after several cytotoxic agents. The most detailed studies, including the examination of the time-course of cell yield change during a 48 h period immediately following treatment, were performed with CY, VCR, FU and $\gamma$-irradiation (Stephens *et al.*, 1977). By pooling these data and those obtained from further experiments during periods when there was no significant change in cell yield (see Stephens *et al.*, 1977 which shows that for all agents the cell yield did not change significantly between 20 and 30 h after treatment) histograms of cell yield were constructed (Fig. 2). The treated distributions were compared with the

TABLE II.—*Effect of various cytotoxic agents on tumour weight cell yield and cell survival*

| Agent | Dose (mg/kg) | Number of observations | Relative tumour weight | P | Relative cell yield/g | P | SF | P | Fraction of surviving cells per tumour | P |
|---|---|---|---|---|---|---|---|---|---|---|
| γ-rays | 940r | 13 | 1·07 | NS | 0·70 | 0·004 | 0·073 | 0·0003 | 0·053 | 0·0003 |
| CCNU | 20 | 9 | 0·84 | NS | 0·70 | 0·004 | 0·0029 | 0·004 | 0·0015 | 0·004 |
| CY | 300 | 11 | 0·92 | NS | 0·69 | 0·001 | 0·0051 | 0·001 | 0·0039 | 0·001 |
| Melphalan | 15 | 7 | 0·97 | NS | 0·66 | 0·02 | 0·011 | 0·02 | 0·0059 | 0·02 |
| BCNU | 35 | 6 | 0·85 | NS | 0·53 | 0·03 | 0·013 | 0·03 | 0·0052 | 0·03 |
| Thio-TEPA | 20 | 6 | 1·05 | NS | 0·76 | 0·03 | 0·26 | 0·03 | 0·22 | 0·03 |
| $HN_2$ | 3 | 6 | 1·30 | NS | 0·83 | 0·03 | 0·93 | NS | 0·91 | NS |
| Actinomycin D | 0·75 | 6 | 0·82 | NS | 1·00 | NS | 1·18 | 0·03 | 1·06 | NS |
| Adriamycin | 15 | 6 | 0·85 | NS | 0·56 | 0·03 | 0·73 | 0·03 | 0·34 | 0·03 |
| Bleomycin | 750 | 6 | 0·95 | NS | 0·69 | 0·03 | 0·65 | 0·03 | 0·41 | 0·03 |
| VLB | 15 | 6 | 0·95 | NS | 0·29 | 0·03 | 0·96 | 0·05 | 0·24 | 0·03 |
| VCR | 3 | 6 | 0·88 | NS | 0·38 | 0·03 | 0·38 | 0·03 | 0·13 | 0·03 |
| Ara-C | 3000 | 6 | 0·87 | NS | 0·57 | 0·03 | 0·80 | 0·03 | 0·39 | 0·03 |
| FU | 250 | 13 | 0·93 | NS | 1·3 | 0·05 | 0·67 | 0·0003 | 0·85 | NS |

B16 Melanomas were treated 20–24 h before assay.
Doses near to $LD_{10}$ were used for all agents, except radiation, and CCNU when about half $LD_{10}$ was used.

well-defined normal distribution obtained from untreated tumours (Fig. 1B).

The distributions of the treated and untreated populations were compared using the Kolmogarov–Smirnov one-sample test (Siegel, 1956), which is relatively insensitive to distribution type. The distributions obtained with all 4 agents were found to be significantly different from the untreated distribution ($P < 0{\cdot}01$). These differences were largely due to differences in the locations of the populations, and we therefore conclude that treatment of B16 melanoma with CY, VCR and γ-irradiation significantly reduced the yield of cells/g and FU treatment significantly increases the cell yield.

Several other agents have been shown to affect cell yield, and the results are summarized in Table II. Each agent was administered at a single dose level near the $LD_{10}$ (except radiation and CCNU; see notes to Table II) and assays were performed 20–24 h later. Relative tumour weight, relative cell yield per gram, SF and *fraction of surviving cells per tumour* were determined. The randomization test for matched pairs (Siegel, 1956) was used to compare the treated and untreated data. The probability that the treated and untreated samples were both drawn from the same population is shown in Table II.

No agent produced any significant effect on tumour weight. However, 4 types of behaviour with respect to cell yield were seen:

(1) The cell yield was greatly reduced (to ~ 30% of control) by VLB and VCR.

(2) The cell yield was reduced to a lesser, though significant extent (to 55–85% of control) by γ-irradiation, CCNU, CY, melphalan, BCNU, thioTEPA, $HN_2$, adriamycin, bleomycin and Ara-c.

(3) The cell yield was not changed by actinomycin D.

(4) The cell yield was significantly increased (by 30%) by FU.

Cell-survival measurements indicated that this tumour is most sensitive to the alkylating agents CCNU, CY, melphalan and BCNU, all of which reduced cell survival by 2 decades or more. Several of the other agents slightly reduced SF, but no significant reduction was demonstrable with $HN_2$ or VLB, and actinomycin D appeared to slightly increase the SF.

### *Cell yield and tumour morphology*

Two groups of agents which produce cell-yield changes have been studied in detail, and in each case a change in tumour morphology has been seen.

*Alkylating agents (CCNU, CY, melphalan, BCNU, thioTEPA, $HN_2$) and γ-irradiation.*—The reduction in cell yield produced by these agents is associated with an increase in cell size. We have measured cell yield/g (recalculated from repopulation curves reported by Stephens & Peacock, 1977) and cell diameters (from cytocentrifuge preparations) at various times after treatment of B16 melanoma with 300 mg/kg of CY (Fig. 3.) and 20 mg/kg of CCNU (Fig. 4). The upper panels of Figs. 3 and 4 show the distribution of cell diameters at several selected

FIG. 3.—Changes in cell yield/g and cell diameter after treatment of B16 melanoma with CY (300 mg/kg). The cell yields were recalculated from data shown in Fig. 6 of Stephens & Peacock (1977). A cytocentrifuge preparation of each cell suspension was prepared, and the diameters of 50 cells were measured. The median diameters and quartiles of each population were calculated, and the full diameter distributions at selected times are shown.

FIG. 4.—Changes in cell yield/g and cell diameter after treatment of B16 melanoma with CCNU (20 mg/kg). The cell yields were recalculated from data shown in Fig. 7 of Stephens & Peacock (1977). For other details see Fig. 3.

times after treatment. In each case there was a gradual increase in mean cell diameter and a concomitant decrease in cell yield/g. At later times the mean cell diameter returned to control levels and cell yield also returned to normal.

Similar studies have been performed for the other alkylating agents up to 48 h after treatment and in each case an increase in cell diameter has been found. If the decreased cell yield is due to the increased cell size, only a small change in cell diameter would be required to markedly reduce yield (25% increase in diameter represents a doubling of cell volume and could lead to a decrease in cells/g of 50%).

*Vinca alkaloids (VLB, VCR).*—These agents appear to produce very rapid lysis of B16 melanoma cells *in vivo*, associated with a large reduction in cell yield. We recently reported that tumours developed large liquefied central regions after exposure to high doses of VCR

(Stephens *et al.*, 1977). We have since seen a similar effect after treatment with VLB, and have compared the fractions of necrosis in histological sections of untreated tumours with those treated for 24 or 48 h with VCR at a dose of 5 mg/kg. The average necrotic fraction measured by point counting increased from $\sim 35\%$ at the time of treatment to 68% by 24 h and 72% by 48 h after drug administration. The similarity between the fraction of necrosis measured 24 and 48 h after treatment correlates well with the time-course of cell-yield reduction reported previously (Stephens *et al.*, 1977). We found that cell yield decreased during the first 24 h after treatment, but then stabilized up to 48 h.

### DISCUSSION

The cell killing effects of cytotoxic agents on solid tumours have been extensively studied using *in vitro* cell survival assays. Two effects may be measured, changes in the yield of cells obtained by dissaggregation of the tissue, and changes in the survival of these cells, indicated by their ability to form colonies of descendants. In many studies, only cell-survival data have been presented and cell yield has been ignored, but we have measured both cell yield and cell survival in the B16 melanoma.

In untreated tumours the mean cell yield/g tissue was $\sim 10^8$, although the tumours apparently contain $3–5 \times 10^8$ cells/g. Thus, the trypsinization appears to release between 20 and 30% of the tumour cells actually present in the tissue. In comparison, Reinhold (1965) estimated that about 10% of the cells in a rat rhabdomyosarcoma were released by trypsinization, and experience with Lewis lung carcinoma in this laboratory suggests that $\sim 10\%$ of the cells in that tumour can be liberated by trypsinization. In our trypsinization procedure cells may be lost in 3 ways: by mechanical damage during chopping and loss during the washes and first trypsinization which was specifically designed to liberate damaged cells, by lysis due to prolonged exposure to trypsin during the main trypsinization, or by removal in the small lumps of undigested tissue filtered out after the trypsinization.

It is generally assumed that the cells obtained by trypsinization are representative of all cells in the intact tumour. Only if there is a significant subpopulation of cells which are either very sensitive or very resistant to trypsinization is this likely to be untrue.

Most of the agents used in this study significantly altered cell yield and cell survival, and two mechanisms which may be associated with cell-yield changes are described. Several alkylating agents and $\gamma$-irradiation caused cell enlargement, a phenomenon which has been observed before with these agents (Castaldi, 1970) and could account for the observed decrease in cell yield/g. A reduction in cell yield has been reported when the rat tumour R1 rhabdomyosarcoma was treated with X-rays or neutrons (Barendsen & Broerse, 1969). The decreased cell yield correlated well with a reduction in cell density estimated by counting cells in histological sections, and may have been due to an increase in mean cell size. Rosenblum *et al.* (1976) have also reported a small reduction in cell yield after treatment of a rat brain tumour with BCNU.

The large change in cell yield produced by the Vinca alkaloids appears to be associated with a rapid and extensive cell lysis within the intact tumour, since there was a doubling of the fraction of necrotic tissue after treatment. Cells treated with VLB or VCR might be more susceptible than untreated cells, to lysis by exposure to trypsin but we feel this is not the major factor leading to reduced cell yield.

In conclusion, our data clearly demonstrate that cell yields can be accurately and reproducibly measured and would play a useful part in the assessment of the antitumour effectiveness of cytotoxic agents.

We thank Dr G. G. Steel for helpful discussion during the course of this work and Dr D. Jones for advice on methods of statistical analysis.

### REFERENCES

ABERCROMBIE, M. (1946) Estimation of nuclear population from microtome sections. *Anat. Rec.*, **94**, 239.

BARENDSEN, G. W. & BROERSE, J. J. (1969) Experimental radiotherapy of a rat rhabdomyosarcoma with 15 MeV neutrons and 300 kV X-rays. *Eur. J. Cancer*, **5**, 373.

CASTALDI, G., ZAVAGLI, G., FIOCCHI, O. & TROTTA, F. (1970) Giant histiocytes after cyclophosphamide. *Experientia*, **26**, 300.

CERIOTTI, G. (1952) A microchemical determination of desoxyribonucleic acid. *J. Biol. Chem.*, **198**, 297.

CHALKLEY, H. W. (1943) Method for the quantitative morphological analysis of tissues. *J. Natl Cancer Inst.*, **4**, 47.

COURTENAY, V. D. (1976) A soft agar colony assay for Lewis lung tumour and B16 melanoma taken directly from the mouse. *Br. J. Cancer*, **34**, 39.

DEHOFF, R. T. & RHINES, F. N. (1961) Determination of number of particles per unit volume from measurements made on random plane sections: the general cylinder and the ellipsoid. *Trans. AIME*, **221**, 975.

DISCHE, Z. (1955) Color reaction of nucleic acid components. In *The Nucleic Acids: Chemistry and Biology 1*. Eds. E. Chargaff & J. N. Davidson, New York: Academic Press, p. 285.

REINHOLD, M. S. (1965) A cell dispersion technique for use in quantitative transplantation studies with solid tumours. *Eur. J. Cancer*, **1**, 67.

ROSENBLUM, M. L., KNEBEL, K. D., VASQUEZ, D. A. & WILSON, C. B. (1976) *In vivo* clonogenic tumour cell kinetics following 1,3-bis (2-chloroethyl)-1-nitrosourea brain tumour therapy. *Cancer Res.*, **36**, 3718.

SCHMIDT, G. & THANNHAUSER, S. J. (1945) A method for the determination of desoxyribonucleic acid, ribonucleic acid, and phosphoproteins in animal tissues. *J. Biol. Chem.*, **161**, 83.

SIEGEL, S. (1956) *Nonparametric Statistics for the Behavioral Sciences*. N.Y. McGraw-Hill, p. 88.

STEPHENS, T. C. & PEACOCK, J. H. (1977) Tumour volume response initial cell kill and cellular repopulation in B16 melanoma treated with cyclophosphamide and 1-(2-chloroethyl)-3-cyclohexyl-1-nitrosourea. *Br. J. Cancer*, **36**, 313.

STEPHENS, T. C., PEACOCK, J. H. & STEEL, G. G. (1977) Cell survival in B16 melanoma after treatment with combinations of cytotoxic agents: lack of potentiation. *Br. J. Cancer*, **36**, 84.